# Atherosclerotic plaque instability in symptomatic non-significant carotid stenoses

**DOI:** 10.1016/j.jvssci.2025.100280

**Published:** 2025-01-17

**Authors:** Paul Cyréus, Katarina Wadén, Sofie Hellberg, Otto Bergman, Mariette Lengquist, Eva Karlöf, Andrew Buckler, Ljubica Matic, Joy Roy, David Marlevi, Melody Chemaly, Ulf Hedin

**Affiliations:** a Department of Molecular Medicine and Surgery, Karolinska Institutet, Stockholm, Sweden; b Department of Vascular Surgery, Karolinska University Hospital, Stockholm, Sweden

**Keywords:** Atherosclerotic plaque instability, Degree of stenosis, Hypoxia, Stroke risk, Symptomatic carotid stenosis

## Abstract

**Objective:**

Carotid endarterectomy for symptomatic carotid stenosis is recommended for patients with >70% stenosis, but not in those with <50%. Because non-significant, low-degree stenoses may still cause strokes, refined risk stratification is necessary, which could be improved by assessing biological features of plaque instability. To challenge risk-stratification based on luminal narrowing, we compared biological features of carotid plaques from symptomatic patients with low-degree (<50%) vs high-degree (>70%) stenosis and explored potential mechanisms behind plaque instability in low-degree stenoses.

**Methods:**

Endarterectomy specimens were taken from symptomatic patients with high-degree (n = 204) and low-degree (n = 34) stenosis, all part of the Biobank of Karolinska Endarterectomies. Patient demographics, image-derived plaque morphology, and gene expression analyses of extracted lesions were used for comparisons. Plaque biology was assessed by transcriptomics using dimensionality reduction, differential gene expression, and gene-set enrichment analyses. Immunohistochemistry was used to study proteins corresponding to upregulated genes.

**Results:**

The demographics of the two groups were statistically similar. Calcification, lipid-rich necrotic core, intraplaque hemorrhage, plaque burden, and fibrous cap thickness were similar in both groups, whereas the sum of lipid-rich necrotic core and intraplaque hemorrhage was higher (*P* = .033) in the high-degree stenosis group. Dimensionality reduction analysis indicated poor clustering separation of plaque gene expression in low-compared with high-degree stenosis lesions, whereas differential gene expression showed upregulation of hypoxia-inducible factor 3A (log_2_ fold change, 0.7212; *P* = .0003), and gene-set enrichment analyses identified pathways related to tissue hypoxia and angiogenesis in low-degree stenoses. Hypoxia-inducible factor 3-alpha protein was associated with smooth muscle cells in neo-vascularized plaque regions.

**Conclusions:**

Plaques from symptomatic patients with non-significant low-degree carotid stenoses showed morphologic and biological features of atherosclerotic plaque instability that were comparable to plaques from patients with high-degree stenoses, emphasizing the need for improved stroke risk stratification for intervention in all patients with symptomatic carotid stenosis irrespective of luminal narrowing. An increased expression of hypoxia-inducible factor 3A in low-degree stenotic lesions suggested mechanisms of plaque instability associated with tissue hypoxia and plaque angiogenesis, but the exact role of hypoxia-inducible factor 3A in this process remains to be determined.

**Clinical relevance:**

Carotid plaques from symptomatic patients with <50% stenosis show morphologic and biological features of plaque instability, comparable to high-degree stenosis, which emphasizes the need for improved stroke risk stratification beyond stenosis severity.

## Graphical abstract

**Figure:**
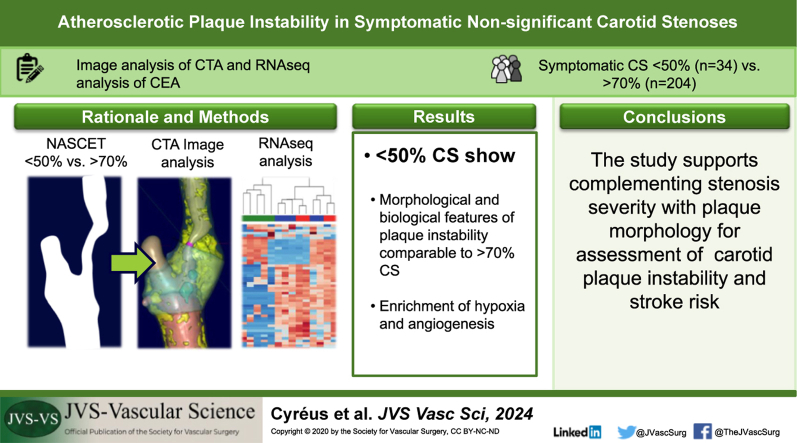

**Article Highlights**

• **Type of Research:** Human study
• **Key Findings:** Investigations of carotid plaques from symptomatic patients with >70% (n = 204) and <50% stenosis (n = 34) by image analysis of computed tomography angiography and analysis of gene expression demonstrated morphologic and biological features of plaque instability in low-degree stenosis, comparable to high-degree stenosis.
• **Take Home Message:** The study emphasizes the need for improved stroke risk stratification beyond stenosis severity.

Atheroembolism from unstable atherosclerotic plaques in the carotid bifurcation is an important cause of ischemic strokes.^1^ Unstable plaques are characterized by inflammation, accumulation of a large lipid-rich necrotic core (LRNC), intra-plaque neovascularization and bleedings (intra-plaque hemorrhage [IPH]), a thin fibrous cap from extracellular matrix degradation and depletion of smooth muscle cells (SMCs), and are generally less calcified (CALC) than stable, asymptomatic lesions.^2^^,^^3^ Risk stratification of patients for stroke-preventive intervention is currently based on results from clinical studies conducted decades ago, where measurements of the degree of stenosis determines recommendations of treatment, including carotid endarterectomy (CEA),^4^ which is largely restricted to patients with high-degree stenosis.^5^, ^6^, ^7^ Nevertheless, as patients with low-degree stenosis below 50% also suffer a significant stroke risk,^8^, ^9^, ^10^, ^11^, ^12^ refined methods for risk assessment are warranted.

Measuring the degree of luminal narrowing in carotid angiograms was the only lesion feature amenable to stratification of carotid stenosis (CS) at the time of the NASCET and ECST trials, which came to serve as surrogate marker for stroke risk.^4^ Subsequent introduction of noninvasive imaging using Doppler ultrasound, computed tomography angiography (CTA), and magnetic resonance imaging (MRI) permits assessment of atherosclerotic plaque features associated with instability and stroke-risk such as plaque volume, thickness, and morphology, which holds promise for improved risk prediction based on lesion biology rather than luminal narrowing.^13^, ^14^, ^15^, ^16^ Of note, the recent European Society for Vascular Surgery guidelines discuss both adding plaque morphology parameters to risk prediction and suggest that CEA can be considered for patients with low-degree (<50%) CS in cases of recurrent symptoms.^5^^,^^6^ There is yet no consensus to introduce plaque morphology assessment for stroke risk stratification in clinical practice.

Utilizing a large biobank of CEA specimens (Biobank of Karolinska Endarterectomies [BiKE]), we have recently demonstrated how molecular signatures of carotid plaques associate with pathophysiologic processes relevant for plaque vulnerability and correlate with both patient and plaque phenotype as determined by plaque morphologic imaging biomarkers.^17^, ^18^, ^19^, ^20^, ^21^, ^22^ Here, this approach was utilized to compare the morphologic and biological features of carotid plaques from symptomatic patients with high (>70%) and low degrees of stenosis (<50%), elucidating for the first time compositional and molecular features of plaque instability in non-significant low-degree stenosis, providing a basis for future optimized stroke-preventive intervention moving beyond stenosis grading.

## Materials and methods

### Study cohort and resources

Patients undergoing CEA for CS at the Department of Vascular Surgery, Karolinska University Hospital, Stockholm, Sweden, were consecutively included in the BiKE, and clinical data was recorded on admission, all described in detail previously.^18^ Here, we utilized a sub-cohort of patients in BiKE enrolled between 2000 and 2020. Inclusion criteria were: symptomatic CS; high- (>70%) or low- (<50%) degree CS based on preoperative Doppler ultrasound according to NASCET criteria,^4^ preoperative carotid CTA, and availability of plaque transcriptomic analyses by RNA sequencing (RNAseq). Indications for CEA in patients with low-degree CS were based on local routines with an overall clinical assessment of symptomatology, surgical risk, and CS diagnostics. Qualifying symptoms were determined by a neurologist and defined as transitory ischemic attack (TIA), minor stroke, and amaurosis fugax (retinal TIA). Patients without qualifying symptoms within 3 months prior to surgery, and with atrial fibrillation, were excluded. In addition, patients with a CS of 50% to 69% were excluded to strengthen comparative analyses. A total of 238 patients were enrolled, 204 with high-degree CS and 34 with low-degree CS (Table I). CEAs were collected at surgery, where the plaques were divided transversally by the surgeon at the most stenotic part, the proximal one-half of the lesion used for RNA preparation, and the distal one-half fixed in 4% Zn-formaldehyde and processed for histology.^18^ Human studies from BiKE were approved by the Ethical Review Board and follow the guidelines of the Declaration of Helsinki. All samples and data in BiKE were collected with informed consent from patients. Publicly available transcriptomic microarray, bulk RNAseq, and single-cell RNA sequencing (scRNAseq) datasets from carotid atherosclerosis were used for validation of the results (Maastricht Human Plaque Study [MaasHPS] GSE163154,^23^; GSE120521,^24^; http://plaqview.uvadcos.io/).^25^

**Table I:** Clinical characteristics of the study cohort

	All	>70% CS	<50% CS	*P*-value
No.	238	204	34	
Age group, years				.512
<60	28 (11.8)	23 (11.3)	5 (14.7)	
60-69	72 (30.2)	59 (28.9)	13 (38.2)	
70-79	96 (40.3)	86 (42.2)	10 (29.4)	
>80	42 (17.6)	36 (17.6)	6 (17.6)	
Male gender	151 (63.4)	129 (63.2)	22 (64.7)	1.000
BMI kg/m^2^	26.05 (4.41)	25.95 (4.18)	26.16 (4.65)	.800
Smoking				.016
Yes	146 (61.3)	121 (59.3)	25 (73.5)	
No	72 (30.3)	68 (33.3)	4 (11.8)	
Unknown	20 (8.4)	15 (7.4)	5 (14.7)	
Statin treatment				.634
Yes	181 (76.0)	156 (76.5)	25 (73.5)	
No	51 (21.4)	42 (20.6)	9 (26.5)	
Unknown	6 (2.6)	6 (2.9)	0 (0.0)	
Hypertension				.427
Yes	163 (68.5)	139 (68.1)	24 (70.6)	
No	51 (21.4)	46 (22.6)	5 (14.7)	
Unknown	24 (10.1)	19 (9.3)	5 (14.7)	
IHD				.727
Yes	148 (62.3)	126 (61.8)	22 (64.7)	
No	88 (36.9)	76 (37.3)	12 (35.3)	
Unknown	2 (0.8)	2 (0.9)	0 (0)	
Diabetes				.102
Yes	58 (24.4)	54 (26.5)	4 (11.8)	
No	180 (75.6)	150 (73.5)	30 (88.2)	
Unknown	0 (0)	0 (0.0)	0 (0.0)	
PAD				.624
Yes	8 (3.4)	7 (3.4)	1 (2.9)	
No	206 (86.6)	178 (87.3)	28 (82.4)	
Unknown	24 (10.1)	19 (9.3)	5 (14.7)	
CKD				.364
Yes	7 (2.9)	7 (3.4)	0 (0.0)	
No	207 (87.0)	178 (87.3)	29 (85.3)	
Unknown	24 (10.1)	19 (9.3)	5 (14.7)	

*BMI,* Body mass index; *CKD,* chronic kidney disease; *CS,* carotid stenosis; *IHD,* ischemic heart disease; *PAD,* peripheral artery disease.

Data is presented as number (%) or mean (standard deviation).

### Computed tomography angiography and image analysis

Carotid CTA was performed preoperatively as previously described.^19^ Reconstructed images (0.625 mm) were analyzed by blinded trained technicians outside of the study team using the ElucidVivo (Elucid Bioimaging Inc) software as previously described.^19^, ^20^, ^21^^,^^26^^,^^27^ Image analyses were available for nine patients with low-degree CS and 48 patients with high-degree CS. In brief, the external carotid artery was excluded from analyses, and the lumen and wall of the common and internal carotid artery were evaluated automatically where the software creates three-dimensional segmentations with voxel-wise discrimination of intraplaque tissue types including LRNC, CALC, IPH, and extracellular matrix (MATX, representing plaque tissue not identified as any of the previous components). The proportion of these components relative to the total wall volume of the plaque was quantified (VolProp), together with structural features such as plaque burden volume ratio (wall volume divided by vessel volume inclusive of lumen and wall) and minimal fibrous cap thickness (smallest measurement of fibrous cap from LRNC to lumen across all cross-sections of the target lesion in μm).

### Carotid plaque gene expression analysis

RNA was prepared from CEA specimens retrieved at surgery using Qiazol Lysis Reagent (Qiagen) and purified by RNeasy Mini kit (Qiagen), including DNase digestion. The concentration was measured using QiaExpert (Qiagen) and quality estimated by a Bioanalyzer capillary electrophoresis system (Agilent Technologies). The library for bulk RNAseq of RNA from plaques was prepared using TruSeq stranded total RNA with RiboZero Globin treatment (Illumina Inc). Paired-end 150 bp read length, NovaSeq 6000 system, S4 flow cells, and v1 sequencing was used for sequencing at 20 mreads/sample. Libraries that yielded less data than aimed for were re-sequenced on SP flowcell. The Bcl to FastQ conversion was performed using bcl2fastq_v2.20.0.422 from the CASAVA software suite, followed by downstream analysis. The raw fastq files were processed using nf-core RNA seq pipeline. Human reference genome GRCh38 obtained from Ensembl was used for alignment. Gene-level counts data of protein-coding and lincRNA genes were considered for all the downstream analysis.

### Histologic and immunohistochemical analysis

Paraffin-embedded cross-sections (5 μm) of CEA specimens from low-degree CS were processed for Masson’s Trichrome and immunohistochemistry staining. In brief, tissue sections were deparaffinized in Histoclear (Histolab) and rehydrated in graded ethanol. For antigen retrieval, slides were subjected to high-pressure boiling in DIVA buffer (pH 6.0; BIOCARE Medical). After blocking with Background Sniper and Peroxidazed 1 (BIOCARE Medical), primary antibodies corresponding to the following proteins and cell markers of interest were used: hypoxia-inducible factor 3 alpha (HIF3-alpha), hypoxia-inducible factor 1 alpha (HIF1-alpha), endoglin (ENG) (all from Sigma, Merck SA), von Willebrand factor, CD68, and alpha-smooth muscle actin (SMA) (all from DAKO, Agilent Technologies). Mouse and rabbit polymer negative control serum were used as control (BIOCARE Medical). Primary antibodies were diluted in Da Vinci Green solution (BIOCARE Medical), applied on slides, and incubated at room temperature for 1 hour. A double-stain probe-polymer system containing alkaline phosphatase and horseradish peroxidase was applied, with subsequent detection using Warp Red (BIOCARE Medical) and Vina Green (BIOCARE Medical). Slides were counterstained with hematoxylin QS (Vector Laboratories), dehydrated, and mounted in Pertex (Histolab). Images were taken with Olympus VS200 slide scanner and processed with the OlyVIA V3.3 software.

### Spatial transcriptomics analysis

A total of six CEA specimens from patients with symptomatic CS with high histologic quality were subjected to spatial transcriptomics analysis using GeoMx Digital Spatial Profiler – Whole Transcriptome Atlas (NanoString), which enables the identification of over 18,000 transcripts in specific preselected areas within the tissue. In brief, paraffin-embedded cross-sections were stained with fluorescent antibodies against SMA and CD68, with DAPI used for nuclear staining (all from DAKO). Based on this staining, regions of interest (ROIs) were manually selected including fibrous cap, LRNC with inflammatory cells, remnants of the media, and neovessels to capture relevant plaque cell types for subsequent analysis with a resolution of 10 μm. DNA indexing oligonucleotide probes with an ultraviolet (UV)-cleavable linker were used to hybridize with the target RNA via *in situ* hybridization. Once each ROI was compartmentalized, UV light was used to release a specific barcode from the ROI of interest and UV-cleaved indexing oligonucleotides collected. Libraries were prepared according to the manufacturer’s instructions, sequenced, counted, and data analyzed using the GeoMx DSP Analysis Suite. In brief, ROI segments were subjected to technical QC (number of read counts, alignment 80%, and sequencing saturation) and biological QC (ROIs with >5% of genes were detected and retained together with genes above the limit of detection in >5% of the retained segments). Next, a quartile three data normalization was performed to allow downstream data analysis. Finally, ROIs were annotated based on biological relevance followed by statistical comparision across six patients using the Student *t*-test.

### Statistical and bioinformatic analysis

Statistical and bioinformatic analyses were performed using R (Version 4.2.2, R Foundation for Statistical Computing) or GraphPad Prism (Version 10). A two-sided *P*-value of less than .05 was considered statistically significant across all tests. Demographics and clinical characteristics were stratified by the degree of stenosis using the ‘tableone’ package in R or using a *t*-test in Prism. Continuous variables were summarized as means ± standard deviation or medians with interquartile ranges, whereas categorical variables were presented as frequencies and percentages. Group differences were assessed using the χ^2^ or Fisher exact tests for categorical variables and *t*-tests or Wilcoxon rank-sum tests for continuous variables. The structure and clustering of high-dimensional gene expression data was visualized using dimensionality reduction techniques principal component analysis, t-distributed stochastic neighbor embedding, and uniform manifold approximation and projection. Differential gene expression analysis was conducted to identify genes significantly associated with high- vs low-degree CS using the ‘limma’ package v.3.53.2. A design matrix was constructed to model stenosis severity as a categorical variable, with samples categorized into high- and low-stenosis groups as previously defined. Specifying a matrix model without an intercept enabled direct comparison between the groups. Linear models were then fitted to each gene, and empirical Bayes smoothing was applied to the standard errors to improve the reliability of the estimates. Multiple testing correction was performed using the Benjamini-Hochberg method. Genes with an adjusted *P*-value below .05 were considered differentially expressed. Functional characterization of differentially expressed genes by gene set enrichment analysis (GSEA) was performed using the ‘enrichR’ package, querying databases Reactome, Gene Ontoology (GO) Biological Process, Kyoto Encyclopedia of Genes and Genomes (KEGG), and MSigDB Hallmark or the ‘cluster profiler’ package querying GO Biological Processes or KEGG.

Plaque morphologic features (volume proportion of LRNC, IPH, CALC, MATX), plaque burden relative to the volume of the entire plaque, and the minimal fibrous cap thickness were compared between high- and low-degree CS using the Wilcoxon rank-sum test. Additionally, a Spearman correlation analysis was utilized to assess the relationships between gene expression profiles and plaque morphologic features, as well as between specific cell markers within the low-degree CS group.

## Results

### Clinical characteristics of study cohort

When comparing patients with high-degree (>70%; n = 204) and low-degree (<50%; n = 34) CS, baseline clinical characteristics were largely similar (Table I). Notably, the low-degree group had a significantly higher proportion of former smokers and fewer non-smokers (52.9% vs 32.4% and 11.8% vs 33.3%; *P* = .016).

### Comparisons between plaque morphology and degree of carotid stenosis

Plaque morphologic features, as assessed by computer-based image analysis of preoperative carotid CTA, was compared between carotid plaques with low- (n = 9) and high-degree (n = 48) CS (see Table II). The sum of LRNC and IPH content was significantly higher in the high-degree CS group (LRNC + IPHVolProp; *P* = .033) and the volume proportion of MATX consequently significantly lower (MATXVolProp; *P* = .016), as this parameter represents plaque tissue not defined as any of the others (CALC, LRNC, IPH). None of the other plaque morphologic features included in the analysis (CALCVolProp, LRNCVolProp, IPHVolProp, PlaqueBurdenVolRatio, and MinMinCapThickness), were statistically different between the two groups (Table II).

**Table II:** Comparison of morphological plaque features from computer-based image analysis of preoperative carotid computed tomography angiography (CTA) as described in methods between <50% and >70% carotid stenosis (*CS*) groups

Variable	>70% CS	<50% CS	*P*-value
No.	48	9	
CALCVolProp	0.03 (0.01-0.08)	0.05 (0.03-0.06)	.200
IPHVolProp	0.09 (0.04-0.12)	0.04 (0.01-0.09)	.058
LRNCVolProp	0.14 (0.09-0.21)	0.10 (0.06-0.13)	.052
MATXVolProp	0.73 (0.67-0.78)	0.81 (0.72-0.87)	.016
LRNC + IPHVolProp	0.23 (0.15-0.31)	0.15 (0.07-0.22)	.033
PlaqueBurdenVolRatio	0.63 (0.54-0.66)	0.56 (0.52-0.59)	.109
MinMinCapThickness	0.35 (0.24-0.44)	0.32 (0.25-0.52)	.598

*CALCVolProp,* Calcified volume as a proportion of total wall volume of the plaque; *IPHVolProp,* intra-plaque hemorrhage volume as a proportion of total wall volume of the plaque; *LRNCVolProp,* lipid-rich necrotic core volume as a proportion of total wall volume of the plaque; *MATXVolProp,* extracellular matrix volume as a proportion of total wall volume of the plaque; *MinMinCapThickness,* smallest measurement of fibrous cap from LRNC to lumen across all cross sections of the target lesion; *PlaqueBurdenVolRatio,* wall volume divided by vessel volume inclusive of lumen and wall.

Data are presented as median (interquartile range).

Median values and interquartile ranges of each parameter compared using Wilcoxon rank sum test with *P* < .05 considered significant.

### Genes associated with hypoxia and angiogenesis are dysregulated in low-degree carotid stenosis

**Fig 1:**
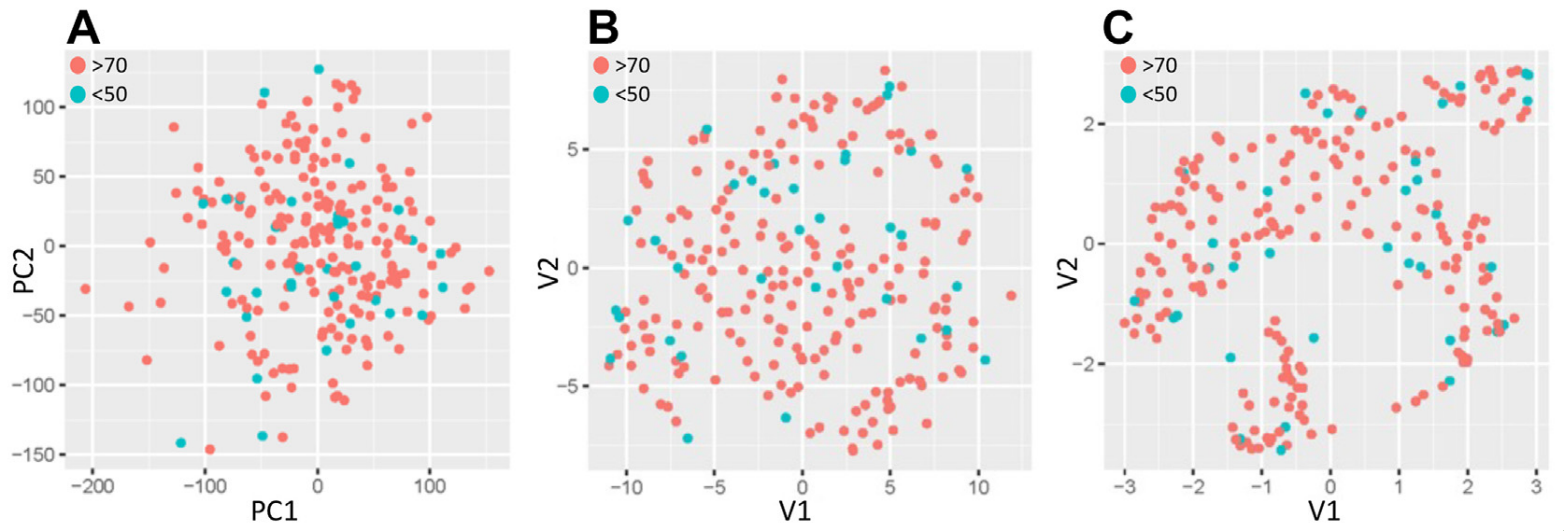
Dimensionality reduction analyses to visualize distribution of gene expression from RNA sequencing (RNA-seq) of carotid endarterectomy (CEA) specimens with <50 % (n = 34) or >70 % (n = 204) carotid stenosis (CS) using Principal Component Analysis (PCA) **(A)**, t–Distributed Stochastic Neighbor Embedding (t-SNE) **(B)**, and Uniform Manifold Approximation and Projection (UMAP) of carotid plaque gene expression from bulk RNAseq data **(C)**. Each point in the scatter plots represents a sample with points colored according to the degree of stenosis for the individual sample. The PCA scatter plot represents the first two principal components (PC1 and PC2) derived from gene expression data and t-SNE and UMAP plots display samples in a two-dimensional space (V1 and V2). Note the absence of clustered data in between the two groups with neither of the analytical methods.

To investigate if biological processes typically associated with atherosclerotic plaque instability were related to the degree of CS, we performed a differential gene expression analysis of RNAseq data from carotid plaques of the cohort (high-degree CS, n = 204; low-degree CS, n = 34). First, the data was transformed for comparison of global gene expression between the two groups by dimensionality reduction analysis using principal component analysis, uniform manifold approximation and projection, and t-distributed stochastic neighbor embedding analyses. Neither linear nor non-linear dimension reductionality methods were able to separate patient clusters with low- or and high-degree CS, indicating similar gene expression signatures between the two groups (Fig 1).

**Fig 2:**
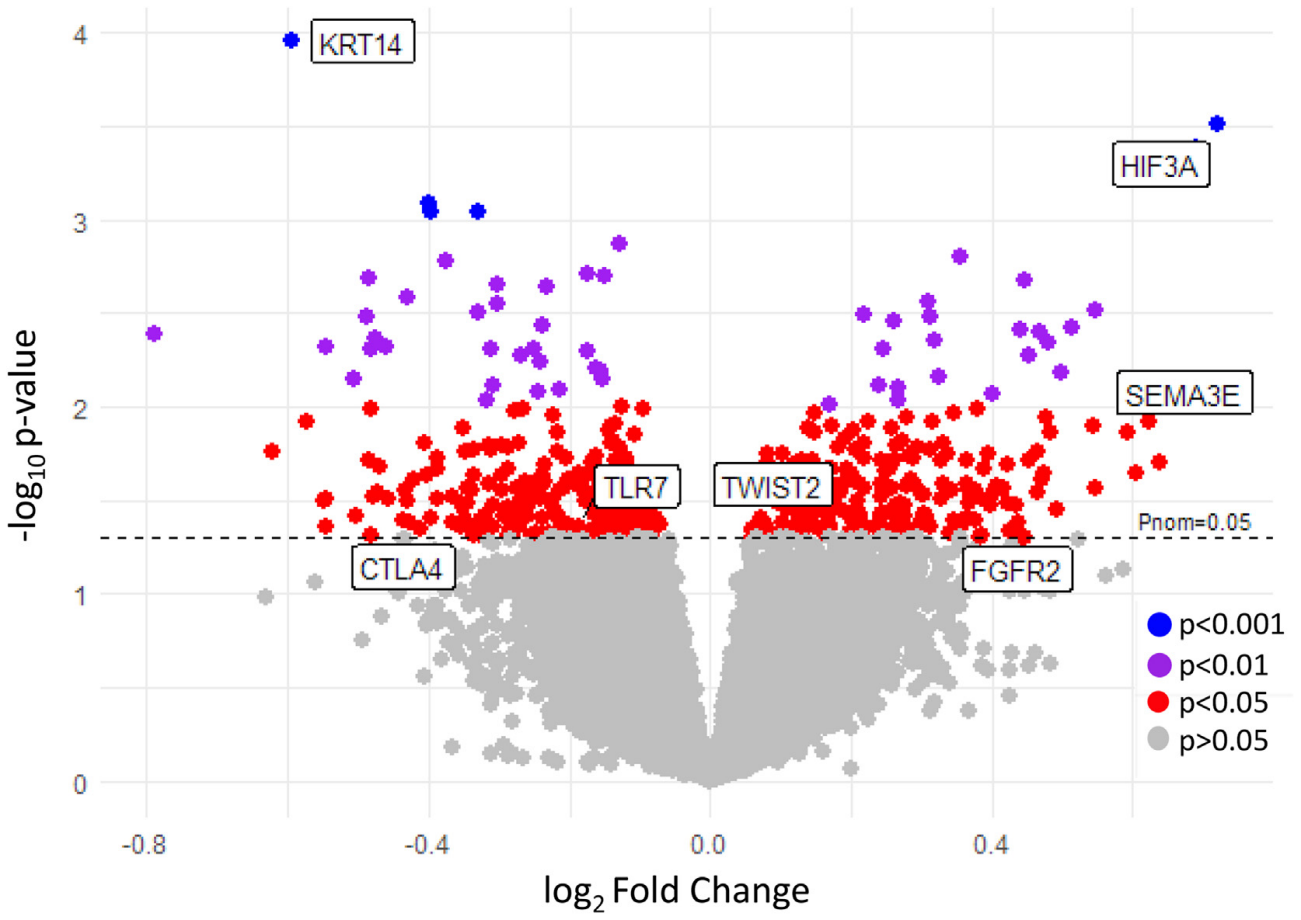
Volcano plot displaying differential gene expression in low-compared with high-degree carotid stenosis (CS) groups. The plot illustrates log_2_ fold changes (x-axis) against −log_10_*P*-values (y-axis) for each gene. Points above the *blue dashed line* represent genes with nominal significance (*P*_nom_ = .05). Genes of particular interest are annotated and highlighted. A positive log_2_ fold change indicates upregulation in <50% stenosis, whereas a negative log_2_ fold change indicates downregulation. *CLTA4*, Cytotoxic T-lymphocyte associated protein 4; *FGFR2*, fibroblast growth factor receptor 2; *HIF3A*, hypoxia-inducible factor 3A; *KRT14*, keratin 14; *SEMA3E*, semaphorin 3E, *TLR7*, toll-like receptor 7; *TWIST2*, Twist Family BHLH Transcription Factor 2.

**Fig 3:**
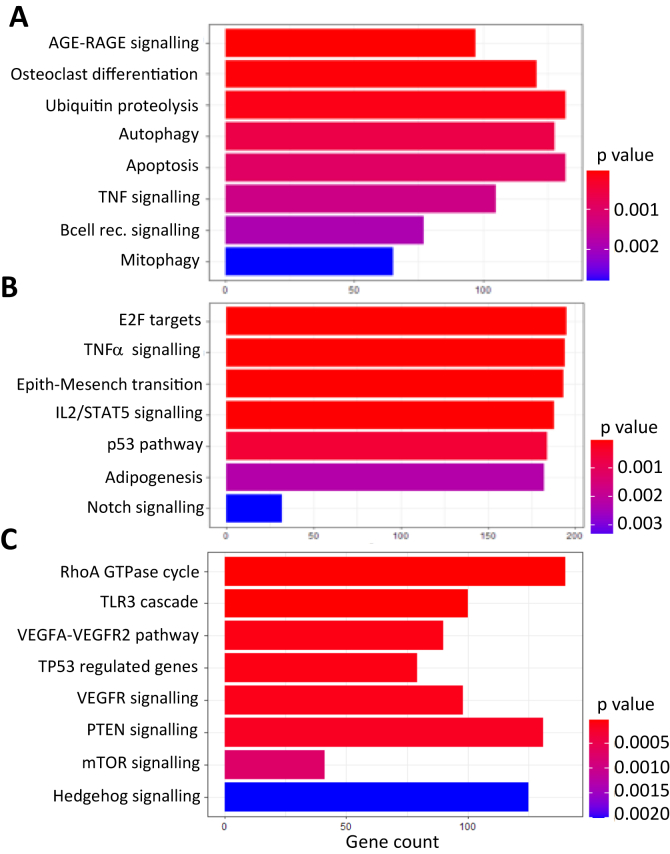
Enriched biological pathways based on differential gene expression observed between low- and high-degree carotid stenosis (CS) groups as determined by different publicly available resources: Kyoto Encyclopedia of Genes and Genomes (KEGG) 2021 Human **(A)**; Molecular Signatures Database (MSigDB) Hallmark 2020 **(B)**; Reactome 2022 **(C)**. Each bar represents a specific biological pathway enriched in low- compared with high-degree CS groups. The y-axis displays the enriched terms, whereas the x-axis displays the gene count. *AGE*, Advanced glycation end product; *IL2*, interleukin 2; *mTOR*, mammalian target of rapamycin; *PTEN*, phosphatase and tensin homolog; *RAGE*, receptor for advanced glycation end product; *STAT5*, signal transducer and activator of transcription 5; *TLR3*, toll-like receptor 3; *TNF*, tumor necrosis factor; *VEGFA*, vascular endothelial growth factor A; *VEGFR2*, vascular endothelial growth factor receptor 2.

Despite similarities, 479 significantly upregulated genes were identified by differential gene expression analysis in low- vs high-degree CS after Benjamini-Hochberg adjustment. In particular, genes related to hypoxia, angiogenesis, and inflammation such as hypoxia-inducible factor 3A (HIF3A), fibroblast growth factor receptor 2 (FGFR2), semaphorin 3E (SEMA3E), and Twist Family BHLH Transcription Factor 2 (TWIST2) were all significantly upregulated in low compared with high-degree CS. In contrast, a few genes related to immune responses such as toll-like receptor 7 and cytotoxic T-lymphocyte associated protein 4, as well as genes related to intermediate filaments (keratin 14), protein metabolism, and ribosomal function, were significantly downregulated in low-degree CS (Fig 2; Supplementary Table I, online only). In addition, GSEA was performed to identify prevalent biological processes in the comparison between low- and high-degree CS utilizing several publicly available ontologies, which showed enrichment of pathways associated with neovascularization, apoptosis, inflammation, cell differentiation/proliferation, tissue remodeling, and metabolic stress (Fig 3).

## Molecular pathways associated with HIF3A expression

**Fig 4:**
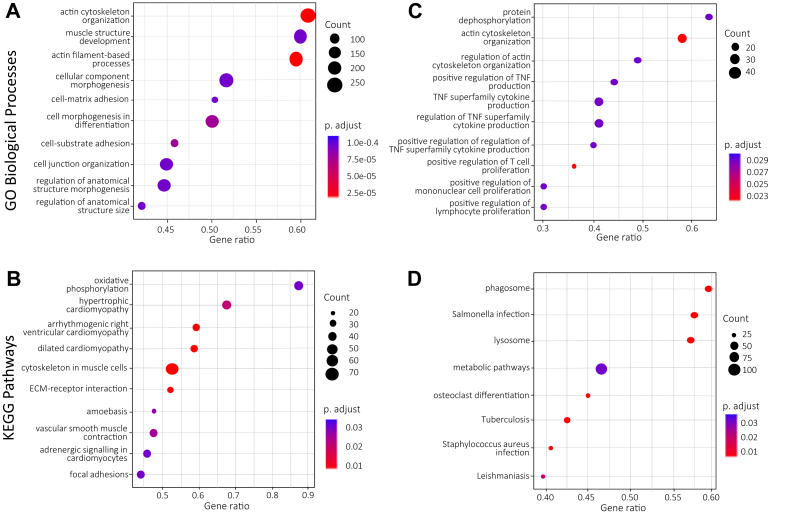
Gene set enrichment analysis (GSEA) of genes positively **(A** and **B)** and negatively **(C** and **D)** correlated with hypoxia-inducible factor 3A (HIF3A) expression in the entire cohort (n = 238) as assessed by Gene Ontology (*GO*) Biological Processes **(A** and **C)** or Kyoto Encyclopedia of Genes and Genomes (*KEGG*) pathways **(B** and **D)**. *ECM*, Extracellular matrix; *TNF*, tumor necrosis factor.

**Fig 5:**
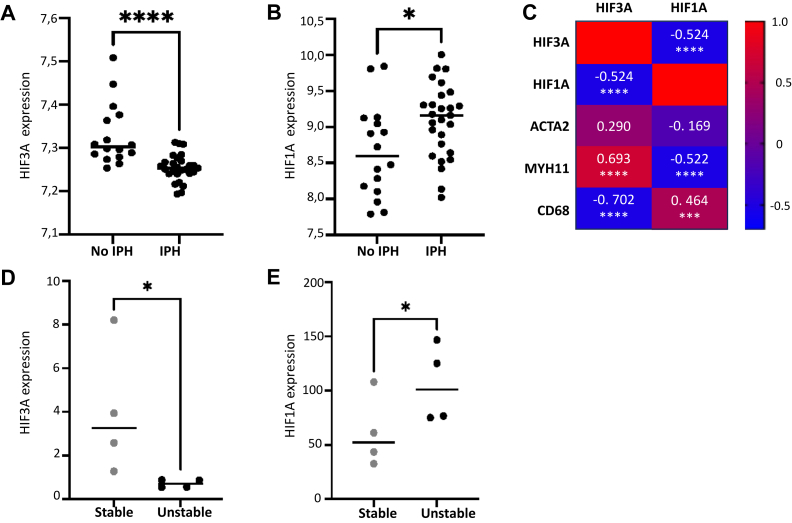
Validation of hypoxia-inducible factor 3A (*HIF3A*) and hypoxia-inducible factor 1A (*HIF1A*) expression in carotid atherosclerotic plaques using two independent cohorts.^23^^,^^24^ HIF3A **(A)** and HIF1A **(B)** gene expression in atherosclerotic plaques with intra-plaque hemorrhage (IPH) (n = 26) or without IPH (no IPH n = 16). Correlation matrix of HIF3A, HIF1A with smooth muscle alpha actin (*ACTA2*), myosin heavy-chain 11 (*MYH11*), and cluster of differentiation 68 (*CD68*) **(C)**.^23^ HIF3A **(D)** and HIF1A **(E)** gene expression in unstable (n = 4) compared with stable (n = 4) atherosclerotic plaque regions.^24^

Because the hypoxia marker HIF3A is not as well-characterized as other members of the HIF family and has previously not been reported in association with atherosclerosis,^28^ we sought to gain further insight into the regulatory pathways associated with HIF3A in atherosclerosis and to explore HIF3A in independent cohorts of patients with carotid atherosclerosis. First, a GSEA performed using GO Biological processes and KEGG Pathways in our entire cohort based on significantly positively correlated genes with HIF3A expression showed enrichment of pathways associated with actin cytoskeleton organization, cell matrix adhesion, and vascular SMC contraction (Fig 4, *A* and *B*; Supplementary Table II, online only). In contrast, a GSEA performed on the significantly negatively correlated genes with HIF3A showed enrichment of pathways associated with inflammation such as tumor necrosis factor, mononuclear/lymphocyte proliferation, and infection (Fig 4, *C* and *D*; Supplementary Table II, online only). Second, we used publicly available microarray and RNAseq data from either vulnerable atherosclerotic plaques with IPH (MaasHPS - GSE163154) or from unstable atherosclerotic plaque regions (GSE120521). In both of these datasets, HIF3A expression in atherosclerosis was confirmed (Fig 5, *A* and *D*), with a decreased expression both in plaques with IPH (Fig 5, *A*) and in unstable vs stable plaque regions (Fig 5, *D*). Conversely, expression of the well-characterized hypoxia marker HIF1A was significantly increased in atherosclerotic plaques with IPH and in unstable vs stable plaque regions (Fig 5, *B* and *E*).

### Expression of marker genes for cells, hypoxia, and angiogenesis in plaques from low-degree carotid stenosis

**Fig 6:**
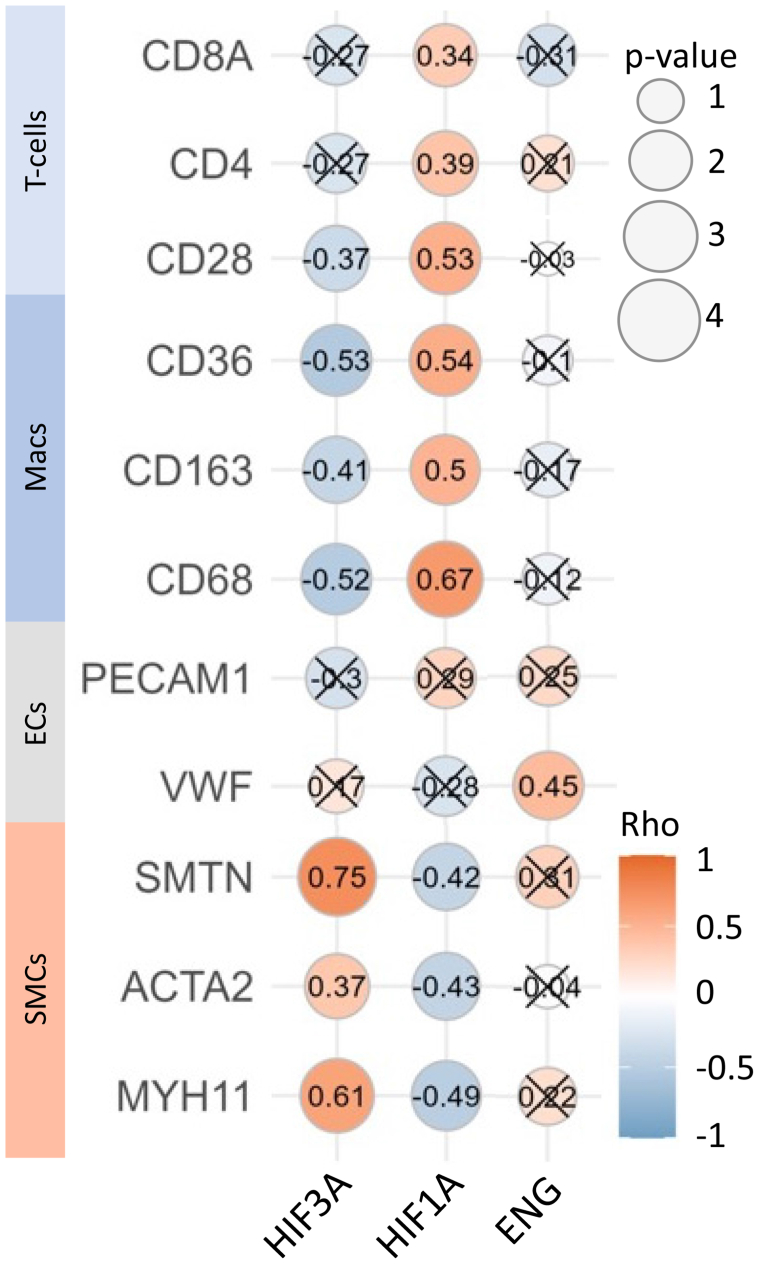
Spearman correlation analysis comparing the expression of cell-specific gene markers for smooth muscle cells (*SMCs*), endothelial cells (*ECs*), macrophages (*Macs*), T-cells with expression of genes associated with hypoxia (hypoxia-inducible factor 1A [*HIF1A*] and hypoxia-inducible factor 3A [*HIF3A*]) and angiogenesis (endoglin [*ENG*]) based on RNA sequencing (RNAseq) data from carotid plaques with low-degree carotid stenosis (CS). Each point represents a gene-marker pair, with the size indicating the significance level (−log_10_ adjusted *P*-value) and the color indicating the strength of the correlation (corresponding numerical value inserted). Data points marked with an X represent a *P*-value > .5 and non-significance. *ACTA2*, Smooth muscle alpha actin; *CD*, cluster of differentiation; *MYH11*, myosin heavy-chain 11; PECAM1, platelet endothelial cell adhesion molecule-1; *SMTN*, smoothelin; *VWF*, von Willebrand factor.

To further characterize the observed upregulation of HIF3A and the enrichment of biological processes related to hypoxia and angiogenesis in low-degree CS, we investigated the correlation between specific cell markers and expression levels of genes associated with hypoxia (HIF1A and HIF3A) and angiogenesis (ENG) (Fig 6). In plaques with low-degree stenosis, a positive correlation was observed between HIF3A and the SMC markers myosin heavy-chain 11 (MYH11; Rho = 0.609, adjusted *P*-value = .0038) and smoothelin (Rho = 0.751; adjusted *P*-value < .001), as suggested by our GSEA where enrichment of SMC associated pathways correlated with HIF3A expression (Fig 4). In contrast, significant negative correlations were observed between HIF3A and the macrophage markers CD68 (Rho = −0.529; adjusted *P*-value = .0134) and CD36 (Rho = −0.529; adjusted *P*-value = .0134). In contrast to HIF3A, HIF1A was negatively correlated with MYH11 (Rho = −0.494; adjusted *P*-value = .0178) and smooth muscle alpha actin (ACTA2; Rho = −0.433; adjusted p-value = 0.0471), but positively correlated with a number of different macrophage and T-cell markers, whereas ENG showed a positive correlation with endothelial cell markers such as von Willebrand factor (Rho = 0.448; adjusted *P*-value = .041) (Fig 6). In support of these findings, similar associations between HIF3A, HIF1A, cellular markers for macrophages, and SMCs were also observed in carotid plaques from the MaasHPS study (Fig 5, *C*).

### Cellular localization of HIF1-alpha and HIF3-alpha in carotid plaques

**Fig 7:**
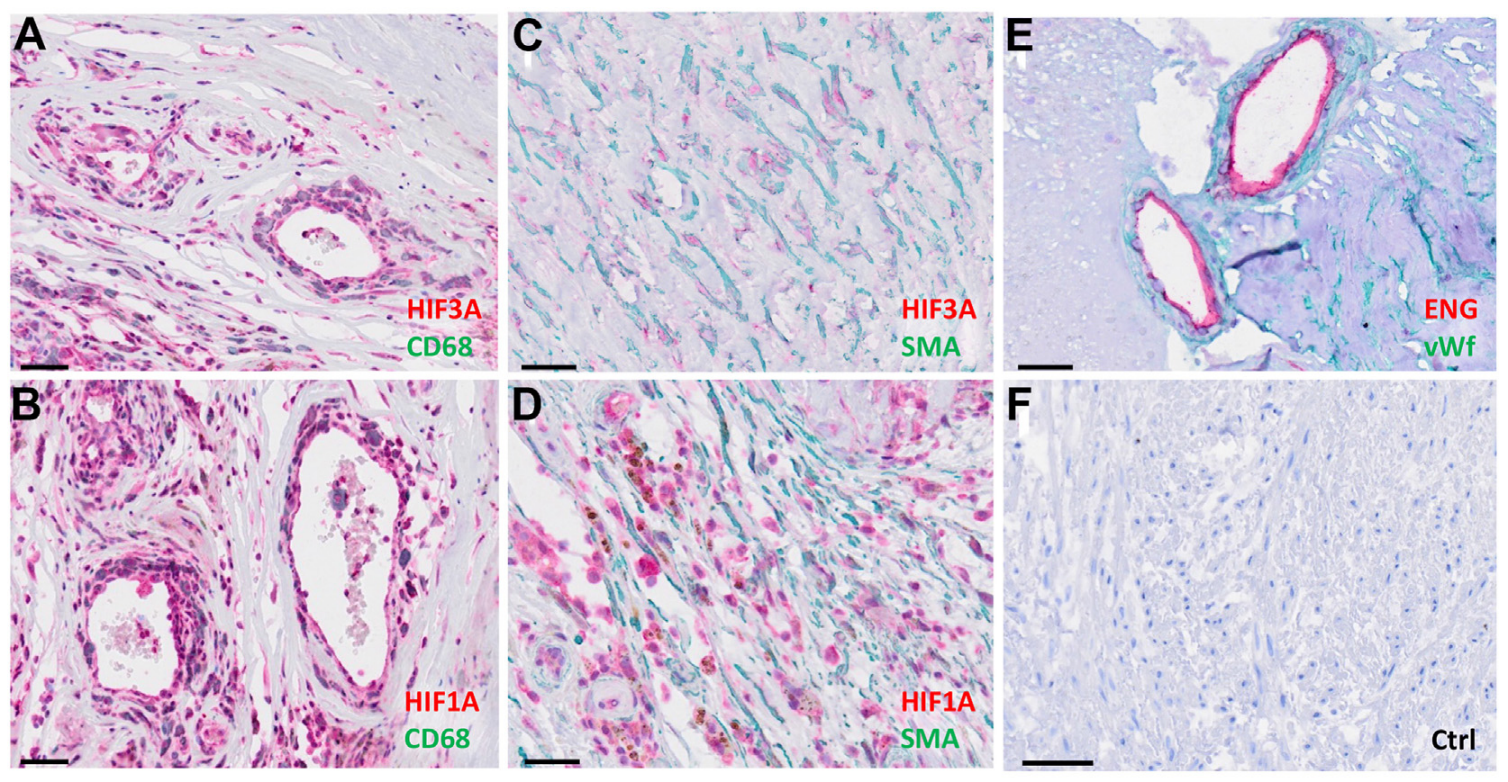
Immunohistochemical double-staining of carotid plaques from low-degree carotid stenosis (CS) lesions localizing hypoxia-inducible factor 3A (*HIF3A*) (**A** and **C**; *Red*) and hypoxia-inducible factor 1A (*HIF1A*) (**B** and **D**; *Red*) to cluster of differentiation 68 (*CD68*)-positive macrophages (**A** and **B**; *Green*) and alpha-smooth muscle actin (*SMA*)-positive smooth muscle cells (SMCs) (**C** and **D**; *Green*), or endoglin (*ENG*) (*Red*) in von Willebrand factor (*VWF*) (*Green*)-positive endothelial cells **(E)**. Control (*Ctrl*) staining **(F)**. Bar indicates 50 μm.

When corresponding proteins were studied by immunohistochemistry of tissue sections from low-degree CS specimens, HIF1-alpha, with a weak double-staining for HIF3-alpha, co-localized in CD68-positive cells in regions with ENG-positive neovessels, indicating association with angiogenesis in the lesions. A distinct staining pattern was seen around SMA-positive cells without co-localization of the two proteins, where HIF3-alpha staining was observed close to SMC nuclei, whereas HIF1-alpha was found in the extracellular matrix surrounding SMA-positive SMCs (Fig 7).

To further explore this finding with respect to the spatial and cellular population context in atherosclerotic plaques, spatial transcriptomics and scRNAseq data were used. HIF3A tended to be expressed higher within SMCs in the fibrous cap area and around neovessels in plaques from six patients (Supplementary Fig 1, online only). Using publicly available scRNAseq datasets from carotid atherosclerotic plaques,^25^^,^^29^^,^^30^ HIF3A was found to be mostly expressed in CD34+ progenitor endothelial cells and in the SMC population, both cell populations with low HIF1A expression. As opposed to HIF3A, HIF1A was strongly expressed in the macrophage/monocyte populations (Supplementary Fig 2, *A* and *B*, online only).

## Discussion

In this study, we investigated mechanisms behind cerebral embolism and symptoms in patients with non-significant, low-degree CS, by comparing morphologic and biological features of carotid plaque instability from symptomatic patients with low- (<50%) vs high-degree (>70%) CS. Image analysis of preoperative carotid CTA showed morphologic features consistent with plaque instability in low-degree CS and comparable with high-degree CS. These findings were supported by analysis of gene expression signatures of CEA specimens demonstrating enriched molecular pathways associated with tissue hypoxia and angiogenesis in plaques from low-compared with high-degree CS, exemplified by SMC-associated upregulation of HIF3A. The results emphasize the need to complement current risk stratification based on stenosis severity with more refined methods that better capture the biology of unstable carotid atherosclerosis.

Image analysis of preoperative CTA from patients with low-degree CS showed morphologic plaque features consistent with atherosclerotic plaque instability and comparable to high-degree CS where only the sum of volume proportions for LRNC and IPH, and consequently MATX, was significantly different between the two groups. Although this finding could suggest more stable lesions in the low-degree CS group,^21^ all patients were symptomatic and assessed eligible for surgery. In addition, the groups were similar with respect to all other features of plaque instability such as volume proportions of LRNC and IPH individually, fibrous cap thickness, and plaque burden. Previously, stroke of undetermined source has been recognized in patients with a mild or non-significant CS together with detection of high-risk plaque features such as IPH, fibrous cap rupture, and inflammation based on both MRI and F^18^-FDG-PET imaging.^11^^,^^31^, ^32^, ^33^ Importantly, if left untreated, such lesions have also been shown to be associated with a significant risk of recurrent stroke or TIA.^8^^,^^10^ Together, these observations emphasize the advantages of including assessment of plaque morphology in stroke prediction using noninvasive imaging methods with the capacity to capture high-risk plaque features beyond stenosis severity.^14^, ^15^, ^16^ Importantly, these arguments were also supported by our observation that gross biological processes reflected atherosclerotic plaque instability and were largely similar between carotid plaques from patients with low- and high-degree CS as assessed by analysis of gene expression signatures from retrieved CEA specimens and dimensionality reduction analyses of RNAseq data using several methods. The relevance and validity of these findings is supported by numerous prior studies from our group linking plaque biology as estimated by transcriptomic signatures to both patient^17^^,^^18^^,^^22^ and plaque phenotype based on imaging biomarkers of plaque (in)stability.^19^, ^20^, ^21^ Of note, these findings were also recognized in the recent European carotid surgery guidelines as arguments for future inclusion of plaque morphology in stroke-risk stratification of patients with carotid atherosclerosis.^6^

Despite similarities in overall transcriptomic profiles, a solid number of differentially expressed genes were identified as upregulated in plaques from patients with low-degree, non-significant CS in comparison to plaques from patients with high-degree CS. To explore potential mechanisms behind plaque instability and symptoms in lesions without clinically significant luminal narrowing, these findings were investigated in more detail. First, GSEA identified significantly enriched molecular pathways associated with inflammation, apoptosis, and angiogenesis, all biological processes previously coupled with plaque instability.^3^^,^^34^, ^35^, ^36^ Interestingly, HIF3A was identified as one of the most upregulated genes, suggesting hypoxic conditions in these lesions despite a low degree of stenosis, but with a plaque burden/volume comparable to those with a high-degree stenosis. This observation was supported by a concomitant upregulation of FGFR2, SEMA3E, and TWIST2, all associated with tissue hypoxia and angiogenesis.^37^, ^38^, ^39^ Previously, tissue hypoxia and induction of the HIF pathway, together with angiogenesis and inflammation, have been demonstrated in carotid atherosclerosis.^28^^,^^40^, ^41^, ^42^, ^43^ As the arterial intima is normally avascular, tissue hypoxia and angiogenesis are expected physiologic responses to plaque enlargement in the intima and ensuing development of plaque instability involving IPH and inflammation.^35^^,^^36^^,^^41^^,^^44^ In support of this hypothesis, atherosclerotic plaque volume has previously been shown to improve stroke risk prediction compared with stenosis severity,^13^^,^^45^^,^^46^ which together would suggest mechanisms whereby large-volume lesions without significant luminal narrowing become vulnerable and at risk for atheroembolism and ischemic stroke.

Although several factors related to hypoxia such as HIF1A and downstream targets of the HIF pathway, such as PDK-1 and VEGF, have been studied in detail in atherosclerosis,^47^ HIF3A expression has not previously been reported. Although expressed at low levels, we observed a significant association between HIF3A expression and SMCs, whereas the correlation with inflammatory cells was negative. In contrast, expression of HIF1A correlated positively with inflammatory cells and negatively with SMCs. These observations were confirmed in two public transcriptomic datasets from carotid atherosclerosis. When interrogating public carotid plaque scRNAseq datasets, HIF3A expression also localized in a CD34+ endothelial cell population in addition to SMCs, confirming our findings. HIF3A has previously been attributed with opposing functions to HIF1A and HIF2A, but there are still controversies to what extent HIF3A actually counteracts the activity of other HIF family members.^48^ Nevertheless, SMCs have been shown to express HIF3A in response to hypoxia *in vitro*,^49^^,^^50^ whereas HIF1A has rather been associated with plaque inflammation and angiogenesis both in the context of hypoxia and lipid metabolism.^51^ These opposing patterns were largely confirmed by immunohistochemistry, where staining for HIF1-alpha and HIF3-alpha seemed to co-localize in CD68-positive cells, but not in SMA-positive SMCs, where nuclear HIF3-alpha staining was seen. Both proteins were found to be expressed in plaque regions with ENG-positive neovessels, further confirming ongoing associations between hypoxia and angiogenesis.^28^^,^^52^ As HIF3A expression was strongly associated with SMC and enrichment of SMC-related biological processes but negatively coupled to IPH both in the MassHPS study and in low-degree compared with high-degree lesions in our cohort, one could speculate whether HIF3A has a role in pro-fibrotic and plaque stabilizing processes rather than those that contribute to instability such as angiogenesis, IPH, and inflammation. However, HIF3A has been suggested to be activated in chronic hypoxia and induces apoptosis when oxygen homeostasis cannot be restored, which would, on the contrary, suggest associations with instability.^48^ Even if our observations suggest a role for hypoxia, possibly triggered by a large plaque burden, in symptomatic low-grade CS, exactly how HIF3A influences pathophysiologic processes related to plaque hypoxia, angiogenesis, and ultimately plaque vulnerability requires further investigations both in human disease and in experimental models.

Even if an unbalanced study cohort with a smaller number of patients with low-degree CS can be seen as a limitation of our study, apart from the BiKE biobank, there are few other resources that can provide detailed molecular analysis from RNAseq data of retrieved CEA specimens coupled with quantified CT imaging data of similar magnitude. Moreover, we cannot exclude that the inclusion of patients in the low-degree CS sub-group was biased from assessment of high-risk plaque features on preoperative CTA as a part of clinical decisions. We believe that such influence cannot be avoided, as imaging of plaque morphology is becoming more and more accepted in clinical practice. Even if analyses performed at the group level demonstrated enrichment of biological processes reflecting plaque instability, we cannot exclude that our cohort also consisted of patients with stable carotid lesions and where symptoms of cerebral embolism originated from other sources. However, as patients with atrial fibrillation were excluded from the study, we minimized analyses of lesions from patients with cardioembolic events, which would be expected to make up a majority of such cases. Finally, assessment of plaque biology was restricted to analysis of gene expression signatures accompanied by bioinformatic analyses and validation using immunohistochemistry and investigations in independent cohorts. Ideally, investigations of corresponding histologic sections would further strengthen plaque analysis. However, we have previously demonstrated a strong association between transcriptomic analyses and patient as well as plaque phenotype as discussed above, in support of the applied analytical strategy.^17^, ^18^, ^19^, ^20^, ^21^, ^22^ In addition, molecular analyses based on bulk tissue preparations do not allow for comprehensive morphologic studies when the plaque has to be separated for the different methods.

## Conclusions

In summary, we demonstrate that plaques from symptomatic patients with non-significant, low-degree CS exhibit morphologic and biological features consistent with atherosclerotic plaque instability, comparable to high-degree CS, where associations between plaque burden/volume, tissue hypoxia, and angiogenesis may be common factors behind plaque instability and symptoms. The study supports complementing measurements of stenosis severity with assessment of plaque morphology for improved stroke risk stratification in patients with symptomatic CS.

## Author contributions

Conception and design: KW, JR, MC, UH

Analysis and interpretation: PC, KW, SH, OB, ML, EK, AB, DM, MC, UH

Data collection: PC, KW, SH, ML, EK, AB, LM, DM, MC, UH

Writing the article: PC, KW, ML, EK, LM, DM, MC, UH

Critical revision of the article: PC, KW, SH, OB, ML, EK, AB, LM, JR, DM, MC, UH

Final approval of the article: PC, KW, SH, OB, ML, EK, AB, LM, JR, DM, MC, UH

Statistical analysis: PC, KW, SH, OB, MC

Obtained funding: UH

Overall responsibility: UH

MC and UH contributed equally to this article and share co-senior authorship.

## Funding

Project funding was obtained from Stockholm County (HMT 20180867), the Swedish Heart-Lung Foundation (20180036, 20200531, 20230447), the Swedish Research Council (2017-01070, 2021-01516), 10.13039/501100004047Karolinska Institutet, King Gustav Vth and Queen Victoria’s Foundation and MedTechLabs.

## Disclosures

A.B. reports shareholder of Elucid Bioimaging.
