# Supplementary material for: Atherosclerotic plaque instability in symptomatic non-significant carotid stenoses
Source: JVS Vasc Sci. 2025 Jan 17;6:100280. doi: 10.1016/j.jvssci.2025.100280 (PMC11874528; doi:10.1016/j.jvssci.2025.100280)
Supplement: Supplementary material [file mmc1.pptx]

## Slide 1
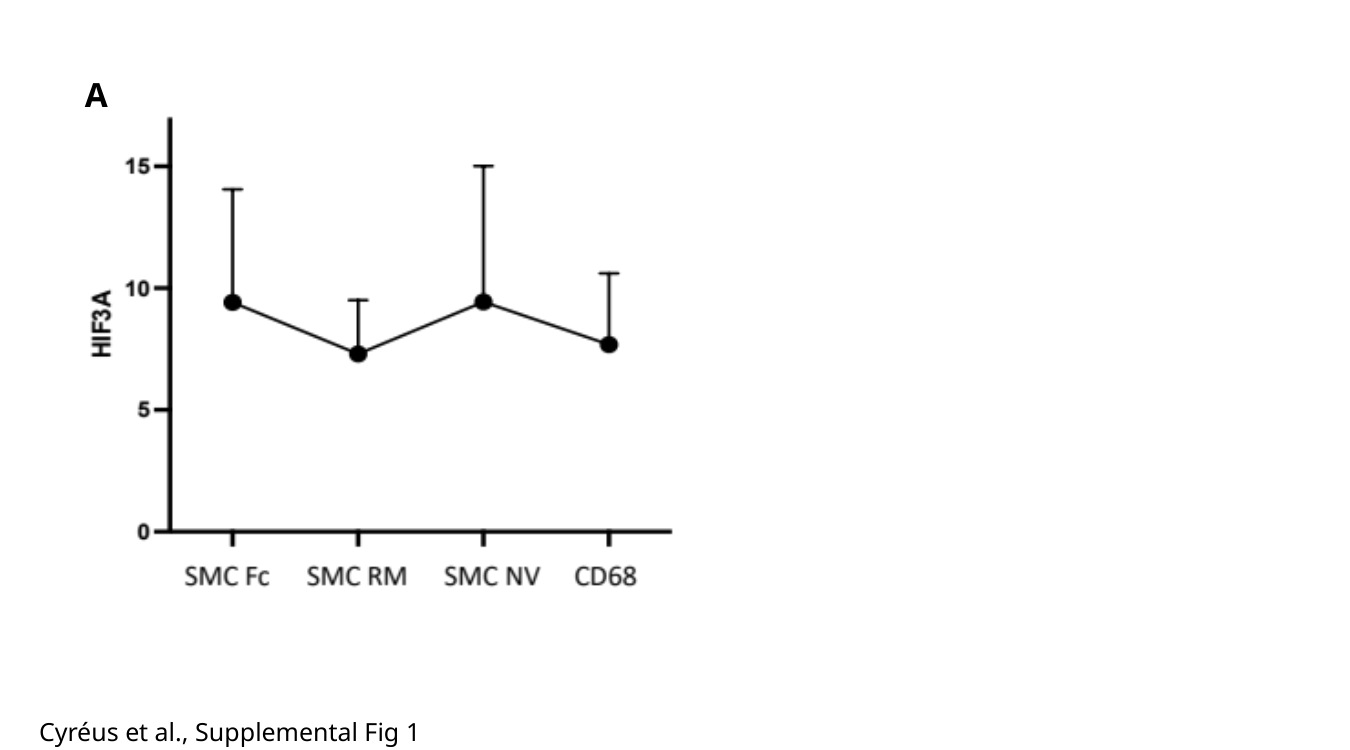

B
A
C
Cyréus et al., Supplemental Fig 1

## Slide 2
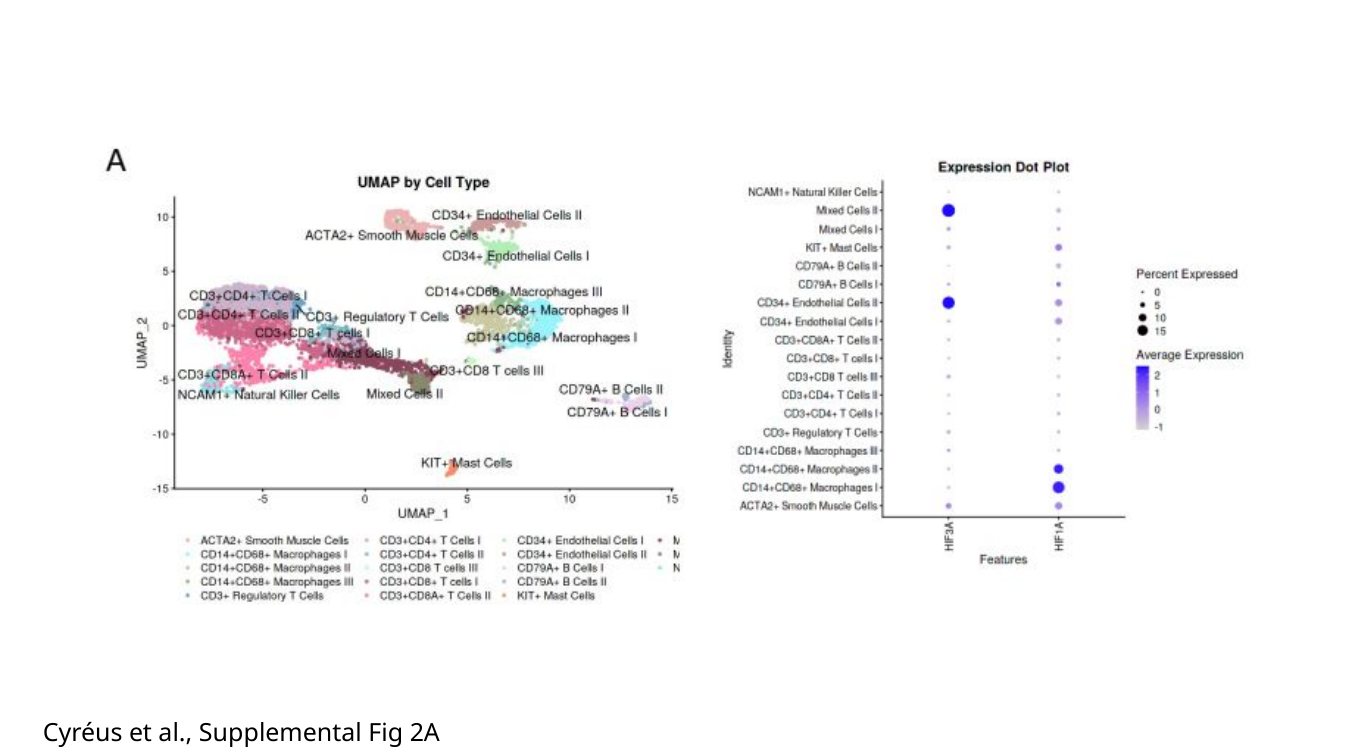

Cyréus et al., Supplemental Fig 2A

## Slide 3
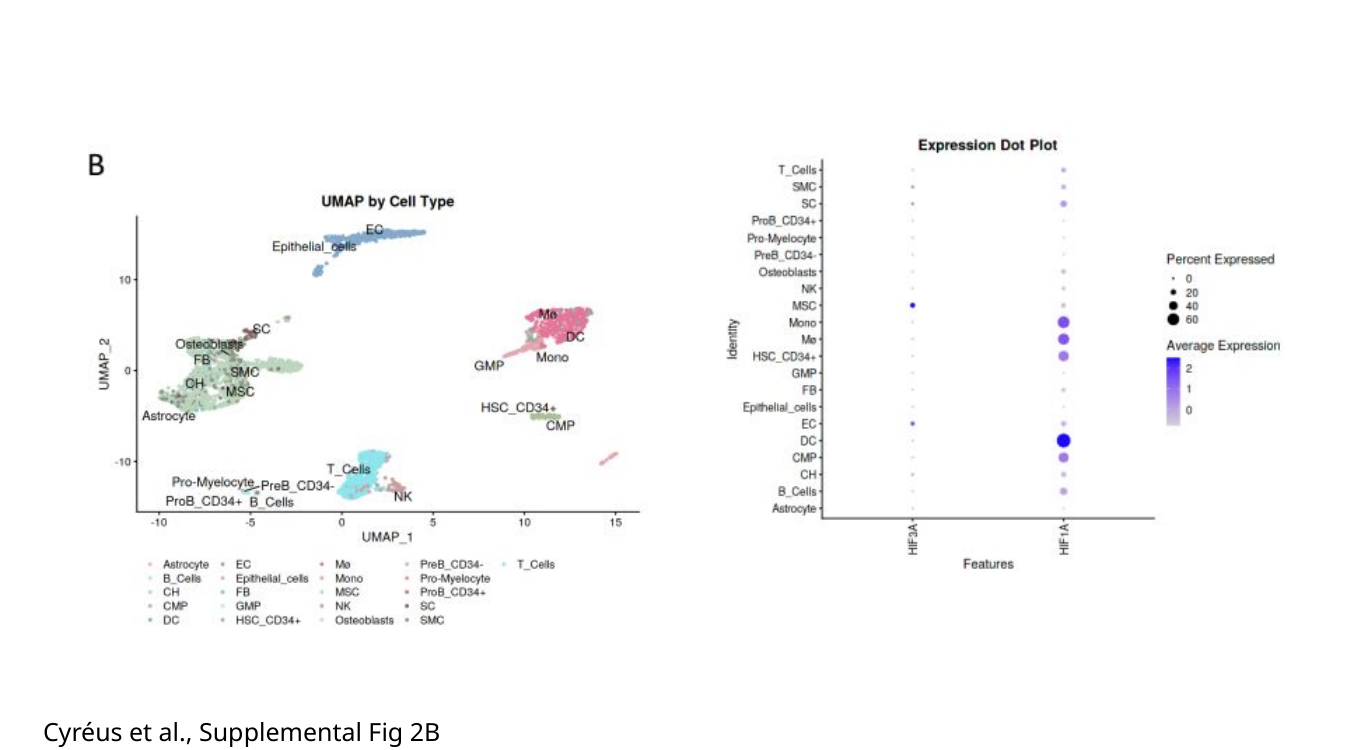

Cyréus et al., Supplemental Fig 2B

## Slide 4
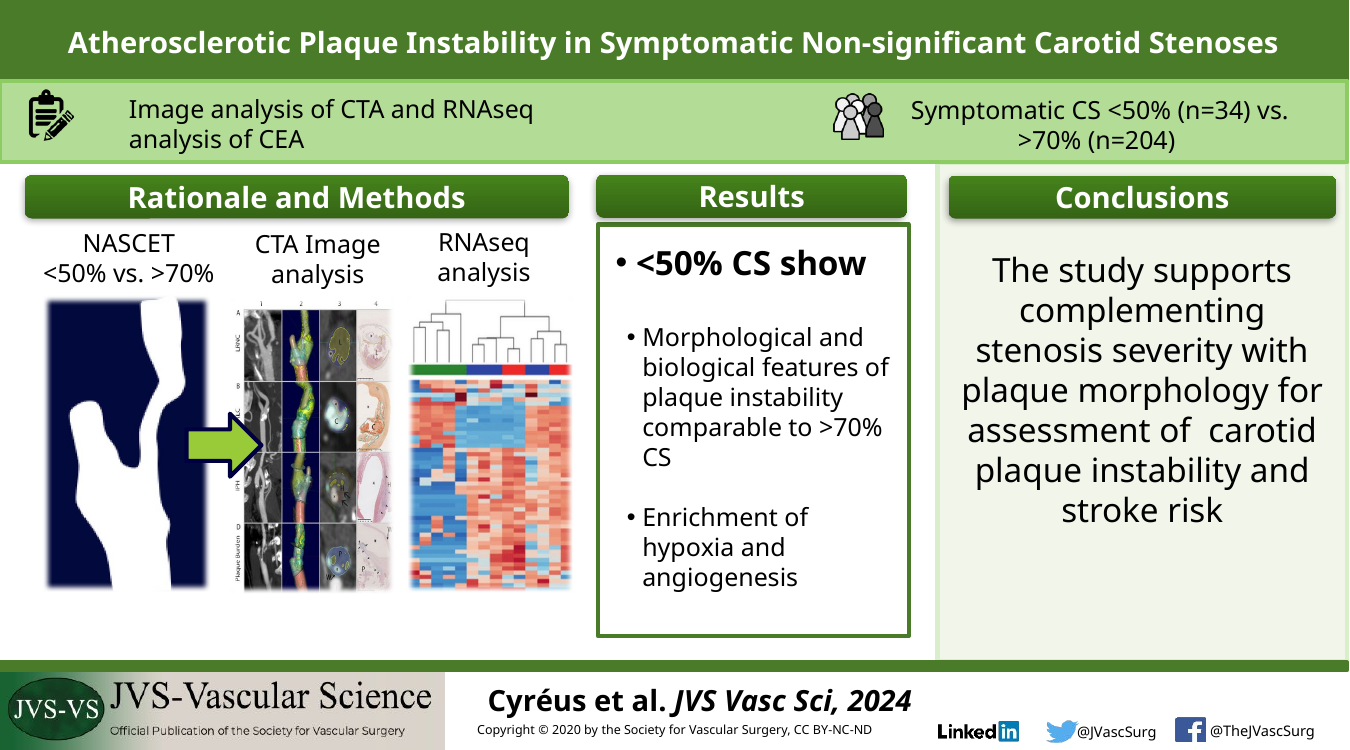

Atherosclerotic Plaque Instability in Symptomatic Non-significant Carotid Stenoses
Image analysis of CTA and RNAseq analysis of CEA
Symptomatic CS <50% (n=34) vs. >70% (n=204)
Results
Rationale and Methods
Conclusions
%
RNAseq
analysis
NASCET
<50% vs. >70%
CTA Image analysis
<50% CS show
Morphological and biological features of plaque instability comparable to >70% CS
Enrichment of hypoxia and angiogenesis
The study supports complementing stenosis severity with plaque morphology for assessment of carotid plaque instability and stroke risk
Cyréus et al. JVS Vasc Sci, 2024
Copyright © 2020 by the Society for Vascular Surgery, CC BY-NC-ND
@TheJVascSurg
@JVascSurg
