# Supplementary material for: Atherosclerotic plaque instability in symptomatic non-significant carotid stenoses
Source: JVS Vasc Sci. 2025 Jan 17;6:100280. doi: 10.1016/j.jvssci.2025.100280 (PMC11874528; doi:10.1016/j.jvssci.2025.100280)
Supplement: Supplementary material [file mmc2.docx]

## Supplemental Tables

**Supplemental Table IA**

**Supplemental Table IB**

**Supplemental Table I**: List of significantly differentially expressed genes in <50% compared with >70% CS. Upregulated genes (A); downregulated genes (B). Genes sorted after p-value. . logFC=log fold change; AveExpr= Average log-transformed expression level of a gene; t= t-statistic derived from a moderated t-test.

**Supplemental Table II**

**Supplemental Table II**: List of the top 100 genes with expression significantly correlated to the expression of HIF3A in the cohort (n=238). Genes sorted after p-value.
